# Tea-inspired curing modulates chemical composition, volatile aroma, and sensory quality of flue-cured tobacco leaves

**DOI:** 10.1038/s41598-026-48595-z

**Published:** 2026-04-14

**Authors:** Yukun Liu, Zhenxie Yi, Xianhai Yang, Keqin Li, Dong Wang, Zhijun Xu, Junfeng Chen, Linjian Dai

**Affiliations:** 1 Hunan Agricultural University, Changsha, 410128 Hunan Province China; 2 Hunan China Tobacco Industry Co., Ltd., Changsha, 410014 Hunan Province China

**Keywords:** Tobacco curing, Tea-inspired curing methods, Sensory evaluation, Volatile organic compounds (VOCs), Polyphenols, Biochemistry, Chemistry, Plant sciences

## Abstract

Curing largely determines the usability and style of tobacco. Drawing on tea processing, we developed three methods using fresh tobacco leaves: a black tea–like process with wilting, rolling, fermentation, and drying (TR), and two green tea key steps that replace rolling with steaming (TZ) or pan-firing (TC) to shape distinct aroma styles, and these were compared with conventional flue-curing (CK). Sensory quality was evaluated by a trained panel and an electronic nose (E-nose). Enzyme activities, macromolecular constituents, major chemical components, and volatile organic compounds (VOCs) were systematically analyzed. The results showed that TR achieved the highest overall sensory performance, with CK and TC comparable, and TZ the lowest. E-nose patterns clearly discriminated the treatments and covaried with aroma scores, reflecting differences in aroma style. Enzymatic reactions induced by TR processing led to a marked decrease in polyphenols, while facilitating macromolecular degradation and promoting the accumulation of key VOCs, including phenylacetaldehyde, indole, and 4,7,9-megastigmatrien-3-one. In contrast, high-temperature pretreatments in TZ and TC rapidly inactivated enzymes before fermentation, leading to higher polyphenol retention but limited macromolecular degradation and key aroma development. Overall, macromolecules are primary negative drivers of sensory quality, whereas chemical balance and key aroma constituents support higher scores. These results indicate that targeted manipulation of key processing steps can effectively direct the formation of tobacco quality and flavor differentiation, while potentially regulating the formation of certain undesirable components. This study provides valuable support for advancing diversified tobacco-curing methods and generating tobacco leaf materials with distinct flavor profiles.

**Supplementary Information:**

The online version contains supplementary material available at 10.1038/s41598-026-48595-z.

## Introduction

Tobacco is an important economic crop, and its quality depends largely on the curing method^1^. Typically, each tobacco type corresponds to a single curing method^2^. For instance, flue-cured tobacco is subjected to flue-curing^3^, while cigar tobacco is air-cured^4^. Although this ensures large-scale production and stable quality, the diversity of tobacco raw materials has been somewhat restricted. With ongoing socio-economic development and changes in consumer preferences, tobacco products have become increasingly diversified, and novel tobacco products are occupying a growing share of the global market^5^. This trend reflects rising consumer demand for differentiated tobacco products and, in turn, places greater demands on the diversity and adaptability of tobacco raw materials^6–8^. Nevertheless, most studies have focused on parameter optimization within established curing methods, and systematic exploration of alternative or entirely new curing processes remains limited^9–13^.

More broadly, tea and tobacco quality are shaped by regulating processing conditions and leaf physical states that influence internal biochemical reactions. Compared with tobacco curing, tea processing offers greater flexibility in combining and adjusting key steps, as reflected in the ability to transform fresh tea leaves into diverse products using different processing techniques^14–16^. Based on this understanding, we hypothesize that fresh tobacco leaves can be processed through multiple innovative curing routes, leading to differences in chemical composition, volatile aroma profiles, and sensory characteristics.

In our preliminary studies, based on the principles of tobacco curing and drawing on black tea processing, we established a curing protocol comprising four key steps—wilting, rolling, fermentation, and drying^17,18^. We demonstrated that this approach significantly increases the levels of reducing sugars and certain aroma constituents, yielding a distinctive aroma profile different from conventional flue-cured tobacco while improving the sensory performance of the leaves. However, rolling-induced cell rupture led to oxidative losses of polyphenolic compounds^19^.

It is well established that polyphenols such as chlorogenic acid, rutin, and scopoletin are key aroma precursors in flue-cured tobacco, and declines in their levels may adversely affect sensory quality^20,21^. Accordingly, we sought to increase polyphenol retention in tea-inspired cured leaves by changing the curing process. Green teas are renowned for their abundant polyphenols and distinctive flavors, which result from two pivotal steps, steaming and pan-firing. These steps rapidly inactivate polyphenol oxidase (PPO) at high temperatures, thereby effectively preserving polyphenols^22–25^. Specifically, steaming quickly denatures enzymes, contributing to a fresh, sweet, and mellow profile, while pan-firing imparts characteristic roasted notes, such as rice, chestnut, and bean-like aromas^26^. Although tobacco leaves differ from tea leaves in botanical characteristics and processing objectives, the basic thermal conditions required for PPO inactivation in leaf tissues are comparable, and the moist-heat effect of steaming and the dry-heat effect of pan-firing may also modulate the volatile aroma composition of tobacco leaves through distinct mechanisms. On this basis, replacing rolling with steaming and pan-firing is expected to substantially improve polyphenol retention, and in turn, broaden the aromatic diversity of tobacco. Purposeful control and the combination of such key steps are also common strategies for shaping the diverse flavor styles of tea^26,27^.

Although sensory evaluation captures multiple aspects of tobacco leaf attributes and subjective usability, it lacks objective quantification. To overcome this, we combine an electronic nose (E-nose) with traditional sensory evaluation to directly assess the utility of novel curing methods by comparing processed leaves in a multidimensional manner^28^. We further compare polyphenol oxidase activity (PPO) and major polyphenol levels to confirm the effectiveness of our process modifications, and examine changes in standard chemical parameters, macromolecular constituents, and volatile aroma compounds to evaluate the global impact on leaf quality. This work provides a basis and a technical pathway for innovating curing techniques and producing high-quality, stylistically diverse tobacco.

## Materials and methods

### Plant material

Middle leaves of Yunyan87 were harvested on June 18, 2024, in Yong’an Town, Liuyang City, Changsha City, Hunan Province (E 113°20′8.46″, N 28°16′47.19″).

### Experiment design and curing method

**Fig. 1:**
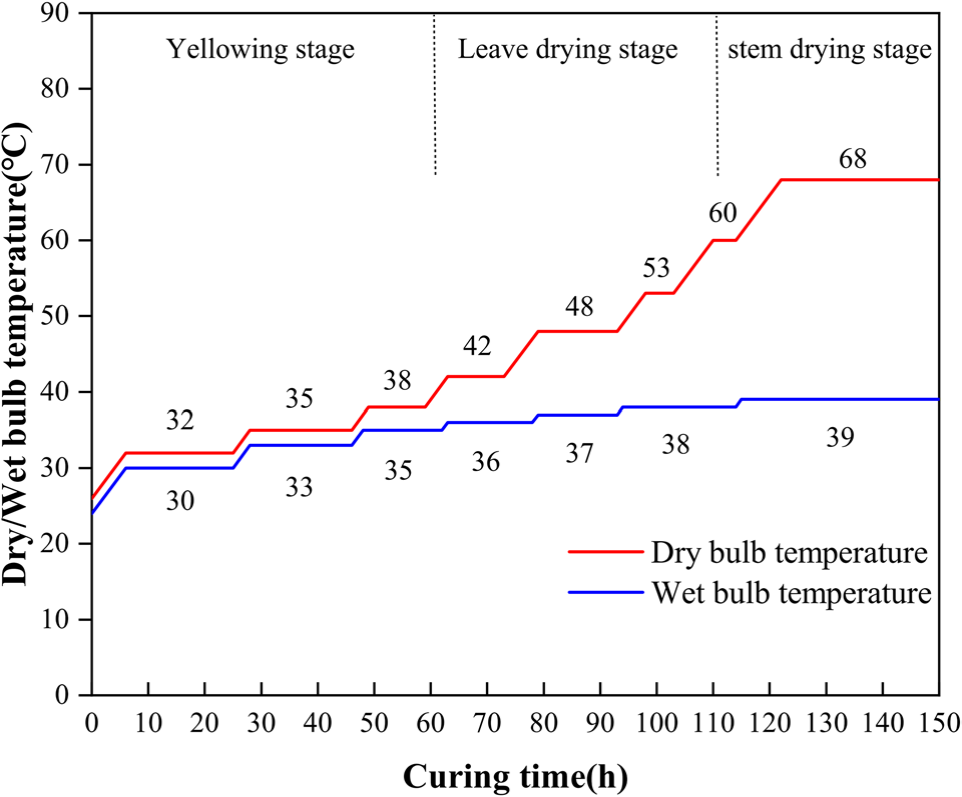
Flue-curing technology.

TR: wilting and cutting, rolling, fermentation, and drying;

TZ: wilting and cutting, steaming, fermentation, and drying;

TC: wilting and cutting, pan-firing, fermentation, and drying;

CK: flue-curing, a three-stage curing process, was employed, consisting of a yellowing stage, a leaf drying stage, and a stem drying stage. During the yellowing stage (0–60 h), the dry-bulb temperature was increased stepwise from 32 ℃ to 38 ℃. Additionally, the wet-bulb temperature was maintained at 30–35 ℃, with heating rates of 1 ℃/h from 32–35 ℃ and 0.5 ℃/h from 35–38 ℃. In the leaf drying stage (60–100 h), dry-bulb temperature was raised from 38 ℃ to 53 ℃ at a rate of 1 ℃/h across the intervals 38–42 ℃, 42–48 ℃, and 48–53 ℃, while the wet-bulb temperature was gradually increased from 35 ℃ to 38 ℃. During the stem drying stage (100–150 h), dry-bulb temperature was further elevated from 53 ℃ to 68 ℃ at 1 ℃/h, and the final temperature was maintained for 20–30 h while wet-bulb temperature was kept at 39 ℃. The detailed time-temperature profile for each stage is shown in Fig. 1.

W (Wilting): Weave fresh leaves into stems, hang them evenly in the wilting machine, and set the dry-bulb temperature to 38 ℃ and the wet-bulb temperature to 36 ℃. Remove the leaves when the moisture content reaches 60.58 ± 2.13 %.

Cutting: Use a knife to remove tissue along both sides of the main vine from the wilted leaves, and cut the lamina into small pieces of approximately 10 cm^2^.

ER (Rolling): Place 2 kg of cut leaves into the rolling machine and roll for 20 min (first 10 min light rolling; next 10 min heavy rolling).

EZ (Steaming): Bring water in a steamer to a boil. Evenly spread 1 kg of cut leaves on a steaming mat, place them in the steamer, and start timing once steam is steadily produced. Steam for 10 minutes. After steaming, remove the leaves and allow them to cool naturally to room temperature before further use.

EC (Pan-firing): Preheat the pan-firing machine. When the pan surface temperature was uniform at 260 °C, quickly add the cut tobacco leaves and stir-fry continuously. Monitor the leaf temperature using a thermometer. Once the leaf temperature reaches 80 °C, maintain the same stir-frying intensity for an additional 2 minutes. After processing, remove the leaves and spread them out to cool naturally to room temperature.

Fermentation: Spread a layer of moist gauze at the bottom of the fermentation frame and evenly place the rolled leaves. The stack thickness is ~5 cm; leave two air holes for heat dissipation and ventilation. Cover with moist gauze to maintain moisture. Ferment at 41 °C for 13 h at 80 % relative humidity.

Drying: Remove the leaves from the frame, unfold them, and place them evenly on a drying tray. Preheat the dryer to 85 °C, dry for 15 min, then equilibrate in a ventilated, dark place for >1 h. Preheat the dryer to 105 °C and dry for 20 min until the moisture content is < 5 %.

**Fig. 2:**
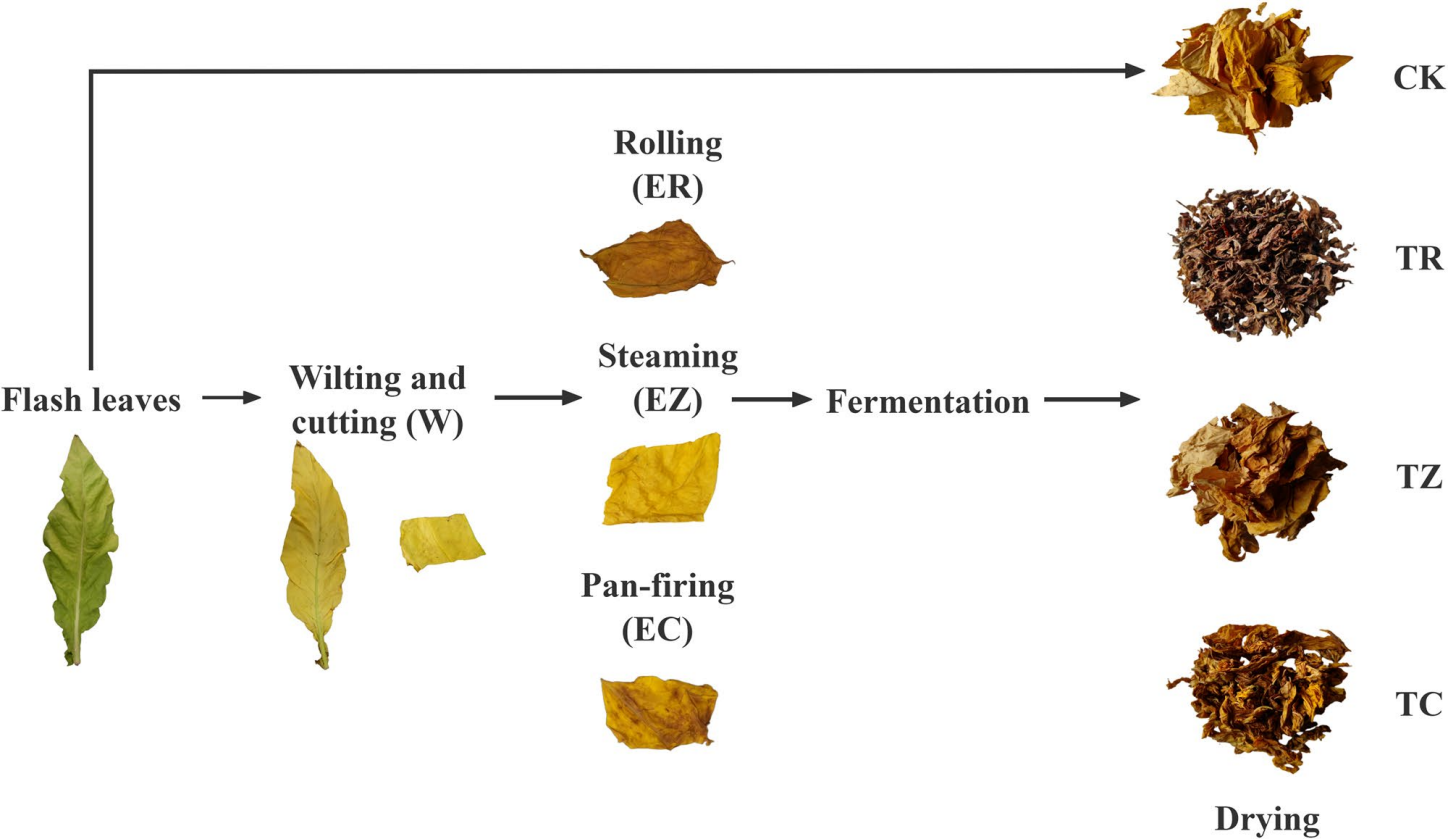
Process flow and treatments for tobacco‐leaf modulation.

Fresh leaves were first wilted and cut (W), then routed into three tea-inspired paths: rolling (ER) → fermentation → TR (black tea–like route); steaming (EZ) → fermentation → TZ (green tea steaming route); and pan-firing (EC) → fermentation → TC (green tea pan-firing route), followed by drying. In parallel, a control batch underwent conventional flue-curing (CK) without the tea-inspired steps. The arrows indicate processing order; the four end products (CK, TR, TZ, TC) were used for subsequent sensory and chemical analyses (Fig. 2).

### Sample collection and preparation

#### Curing and sampling

The sampling sites were open and unobstructed, and the sampled fields were not adjacent to roads or other potential sources of interference. Tobacco plants showed no obvious symptoms of pests or diseases, and border-row leaves were excluded from sampling. Each plant was maintained with 18 retained leaves, and leaves at positions 8, 9, and 10 were selected as experimental materials.

Leaves were harvested at the optimum maturity stage, characterized by softened and whitened midribs, pronounced detachment of surface trichomes, evident leaf yellowing, and an overall appearance of approximately equal proportions of green and yellow coloration. A total of 270 kg of tobacco leaves was harvested.

#### Flue-curing

From the harvest, 60 kg of leaves were allocated to flue-curing. The material was split into three 20 kg biological replicates and cured in the central zone of a barn using the standard three-stage method.

#### Wilting and cutting

The remaining leaves (210 kg) were uniformly wilted. After wilting, the midribs were removed, and the lamina was sliced. From this pool, 300 g of representative material was collected, evenly divided into 6 biological replicates, wrapped in aluminum foil, flash-frozen in liquid nitrogen, and stored at −80 ℃ for enzyme activity detections.

#### Rolling, steaming, and pan-firing

ER, EZ, and EC were each performed in triplicate following the established technical protocols. Immediately after each treatment, 100 g of material per replicate was collected and split into two 50 g biological replicates (n = 6 per treatment for enzyme activity detections). All samples were wrapped in aluminum foil, flash-frozen in liquid nitrogen, and stored at −80 ℃ until analysis.

#### Fermentation and drying

All remaining treated materials then underwent identical fermentation and drying.

### Determination of sensory evaluation

For each treatment, 200 g of cured tobacco conditioned at 22 ± 2 ℃ and 60 ± 5 % RH for ≥48 h was cut into strips and hand-rolled into cigarettes. Paper porosity, rod length/diameter, draw resistance, and target tobacco mass per rod were identical across treatments. Sensory evaluation was conducted by a 7-member certified panel from the Technology Center of China Tobacco Hunan Industrial Co., Ltd. All panelists held official sensory evaluation certificates and had extensive experience in industrial tobacco quality assessment.

Sensory assessments followed a structured 108-point evaluation system routinely applied in industrial practice to assess the usability and quality of tobacco raw materials. Aroma characteristics (36 points)-aroma quality (9), aroma quantity (9), translucency (9), and impurities (9); Smoke characteristics (18 points)-concentration (9) and softness (9); and Taste characteristics (54 points)-strength (9), irritation (9), aftertaste (9), sweetness (9), dryness (9), and cleanness (9). Cigarettes were prepared from treatment-specific strips only; no flavoring or humectants were added. Scores were recorded on individual ballots and aggregated for analysis. Before formal evaluation, panelists underwent routine calibration, and all samples were evaluated in a blinded manner to ensure consistency and reproducibility of sensory results.

### E-nose analysis

To assess the total odor difference among the samples, we made slight modifications to the method originally described by Zhang et al^29^. 5.0 g of each of four different treatment samples was accurately weighed, sealed with parafilm, and equilibrated for 30 min prior to analysis. The E-nose (PEN3, Airsense, Schwerin, Germany) detection was performed using a direct headspace aspiration method, in which the injection needle was inserted into the sealed 50 mL conical flask containing the sample. The operational parameters were set as follows: sampling time, 1 s; sensor self-cleaning duration, 120 s; sample injection time, 5 s; injection flow rate, 600 mL min^−1^; and analytical sampling duration, 75 s. Data acquired between 57 and 60 s were selected for statistical processing and comprehensive analysis. Sensor-specific detection biases are shown in Table 1. The analysis of the experimental samples was conducted thrice.

**Table 1:** E-Nose sensor-specific detection biases.

Number	Sensor	Detection Biases
1	W1C	Aromatic compounds
2	W5S	Nitrogen oxides
3	W3C	Ammonia, aromatic compounds
4	W6S	Hydrogen
5	W5C	Alkanes, aromatic compounds
6	W1S	Methane
7	W1W	Sulfides and terpenes
8	W2S	Alcohols, aldehydes, and ketones
9	W2W	Aromatic components and organic sulfides
10	W3S	Long-chain alkanes

### Determination of enzyme activities

The enzyme activities of polyphenol oxidase (PPO), Peroxidase (POD), α-amylase, and the content of malondialdehyde (MDA) in tobacco leaf samples subjected to different treatments (W, ER, EZ, EC) were measured using commercial assay kits (Beijing Solarbio Science & Technology Co., Ltd.) following the manufacturer’s protocols. The specific kits used were as follows: Polyphenol Oxidase (PPO) Activity Assay Kit (Cat: BC0195), Peroxidase (POD) Activity Assay Kit (Cat: BC0095), α-amylase (α-AL) Activity Assay Kit (Cat: BC0615), and Malondialdehyde (MDA) Content Assay Kit (Cat: BC0025).

### Determination of index

Determination of major chemical constituents following the method described by Chen et al: the content of total sugar, reducing sugar, total alkaloids, chloride ion, potassium, and total nitrogen was measured using the continuous-flow method^30^.

Determination of macromolecular components: the content of plastid pigments, including chlorophyll a, chlorophyll b, and total carotenoids, was determined by applying ethanol-spectrophotometry; the total chlorophyll was calculated as the sum of chlorophyll a and chlorophyll b; the total plastid pigment content was calculated as the sum of chlorophyll a, chlorophyll b, and carotenoids. The protein and starch contents were determined by the continuous-flow method^30^.

Determination of Polyphenols, including chlorogenic acid, rutin, scopoletin, and kaempferol, was measured using high-performance liquid chromatography (HPLC)^31^.

### GC-MS analysis

Volatile organic compounds (VOCs) were extracted from tobacco leaf samples via simultaneous distillation-extraction (SDE).

In total, 5 g of dried tobacco powder, 5 μL of nitrobenzene (internal standard, chromatographic grade), 10 g of anhydrous sodium sulfate, and boiling chips were placed in a 250 mL round-bottom flask with 105 mL of distilled water. The flask was connected to the aqueous phase of the SDE apparatus, and the contents were boiled using a heating mantle. For the organic phase, 150 mL of dichloromethane (analytical grade) was added to a separate 250 mL flask connected to the organic phase port, and the flask was heated in a water bath at 60 °C. Distillation commenced upon condensation, with extraction lasting 2 h. The organic solvent was combined, dehydrated with anhydrous magnesium carbonate, vacuum-filtered, and concentrated to 1 mL at 45 °C using rotary evaporation. The concentrate was filtered through a 0.22 μm organic membrane and stored in vials for analysis.

A gas chromatograph coupled with a mass spectrometer and a SH-I-5Sil capillary column (30 m × 0.25 mm × 0.25 μm) was used for measurement. Helium (>99.999% purity) at a constant flow rate of 1 mL/min was used as the carrier gas. The injection volume was 1 μL, the split ratio was 10:1, and the injector temperature was 250 ℃. Solvents were rinsed twice pre- and post-injection, and samples were rinsed three times. The GC was programmed as follows: Initial: 50 ℃ (held for 2 min); Ramp 1: 4℃/min to 200 ℃ (held for 10 min); Ramp 2: 2 ℃/min to 215 ℃ (held for 5 min); Ramp 3: 0.5 ℃/min to 220 ℃ (held for 2 min); Ramp 4: 2 ℃/min to 300 ℃ (held for 10 min); Total run time: 115 min. The MS parameters were as follows: Ionization mode: electron impact at 70 eV; ion source temperature: 230 ℃; interface temperature: 250 ℃; solvent delay: 6 min; scan range: 40–550 m/z; detector voltage: automatic optimization.

### Qualitative and quantitative determination of volatiles

Volatile compounds were identified by comparing their mass spectra with entries in the NIST 14 mass spectral library and using retention index (RI) information. RI values were calculated according to Eq. (1) based on the retention behavior of compounds on the capillary column and were used to assist compound identification rather than quantification. The RI was calculated using the following formula:

1 $$RI = 100n \times 100\frac{{t^{\prime}\left( i \right) - t^{\prime}\left( n \right)}}{{t^{\prime}\left( {n + 1} \right) - t^{\prime}\left( n \right)}}$$

Quantitative analysis of volatile compounds was performed on a relative basis using the area normalization method^32^. The relative abundance of each compound was calculated as the percentage of its peak area relative to the total peak area of all detected volatile compounds within the same chromatogram.

#### Data statistics

All data were processed using Microsoft Excel 2019 and SPSS 22 (IBM SPSS Statistics, Chicago, USA). Tukey’s honest significant difference (HSD) test at the 95% confidence level was applied to multiple comparisons. Figures were generated using Origin 2021 (OriginLab, Northampton, MA, USA).

Heat maps were generated using Chiplot. Partial least squares discriminant analysis (PLS-DA), variable importance in projection (VIP) analysis, and redundancy analysis (RDA) were conducted using the OmicStudio online platform (https://www.omicstudio.cn)^33^.

## Results

### Sensory evaluation and E-nose analysis

**Fig. 3:**
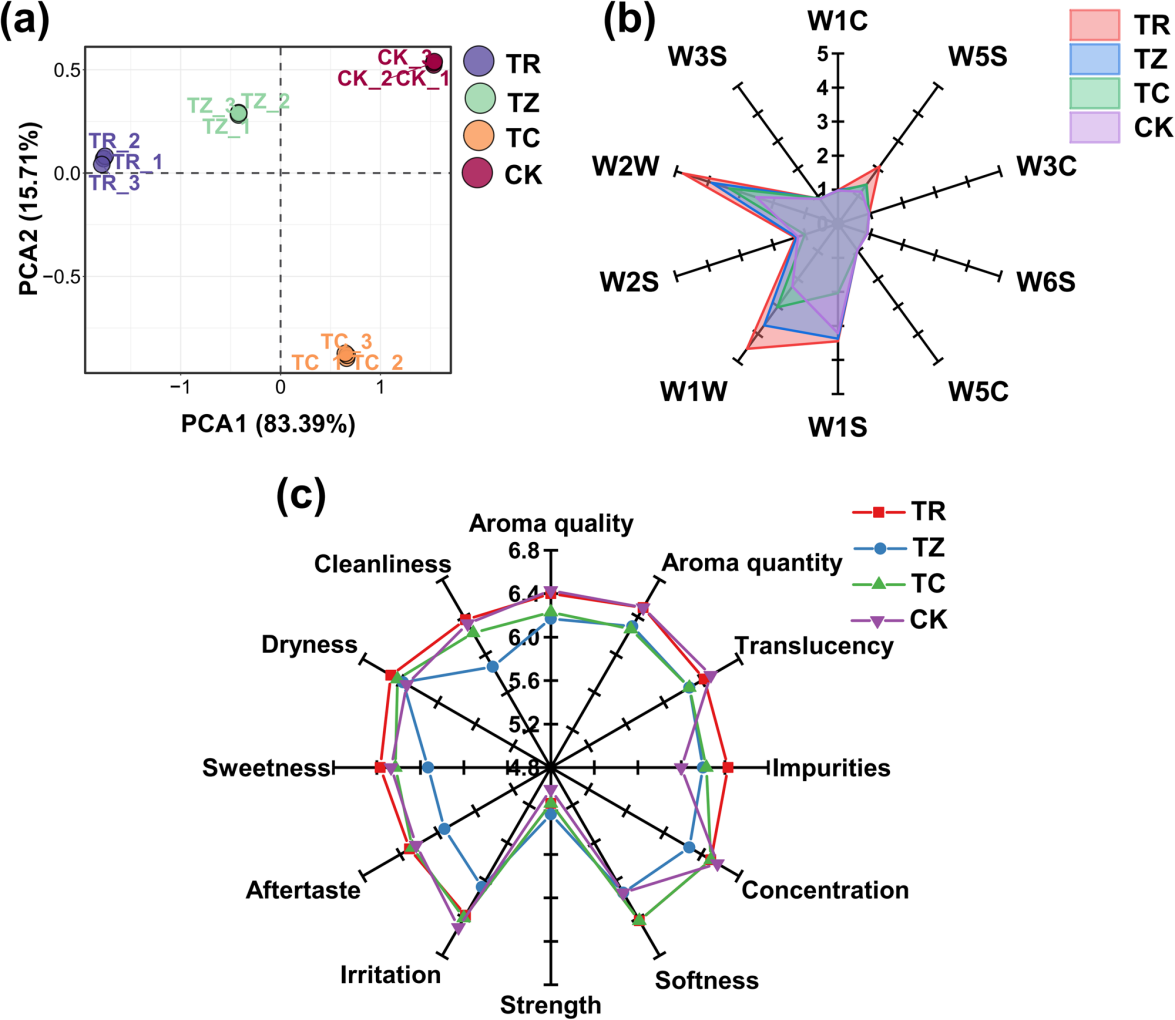
Sensory quality of tobacco leaves with different curing methods. (**a**) PCA of E-nose; (**b**) radar plot from E-nose analysis; (**c**) radar plot of sensory quality evaluation scores, larger enclosed areas in the radar plots indicate better overall sensory performance.

E-nose analysis evaluated the overall odor responses of tobacco leaves subjected to different curing methods. Principal component analysis (PCA; Fig. 3a) separated the samples according to their E-nose profiles. Notably, PC1 and PC2 explained 83.39% and 15.71% of the total variance, respectively, and samples from different curing methods formed distinct clusters.

Radar plots based on the ten sensor signals further illustrated treatment differences (Fig. 3b). Among them, W5S, W1S, W1W, W2S, and W2W showed higher response intensities and larger between-treatment variation. In contrast, W1C, W3C, W6S, W5C, W2S, and W3S exhibited weaker, more similar responses across treatments.

Across sensors with stronger signals, TR generally exhibited the highest responses, followed by TZ and TC, with CK showing the lowest responses (Fig. 3b).

Sensory evaluation directly reflects tobacco usability. As shown in the radar plot (Fig. 3c), TR exhibited the largest sensory profile area among the four treatments, indicating consistently higher scores across multiple evaluated attributes. CK showed a sensory profile close to that of TR, followed by TC. In contrast, TZ had the smallest overall profile area, suggesting that TR achieved the greatest sensory advantage. More specifically, TR scored higher in impurities, sweetness, and dryness. Meanwhile, TZ received lower scores for aroma quality, irritation, aftertaste, sweetness, and cleanliness. TC showed reduced values for aroma quality, aroma quantity, and translucency, but was comparable to TR and CK in concentration and softness. Furthermore, CK was characterized by the lowest impurity scores but exhibited relatively higher translucency, concentration, and irritation.

To capture broader flavor profiles, 12 indices were grouped into three dimensions: aroma (aroma quality, aroma quantity, translucency, impurities), smoke (concentration, softness), and taste (strength, irritation, aftertaste, sweetness, dryness, cleanliness) (Table 2). The total score was highest for TR, with CK and TC performing at statistically similar levels, and TZ was lowest (Tukey, *p* < 0.05). Across the three dimensions, TZ recorded the lowest scores and was significantly lower than TR. Moreover, TC exceeded TZ in smoke and taste, but its aroma score was lower than that of TR. CK showed a higher taste score than TZ and, for aroma, smoke, and taste, did not differ significantly from TR or TC. These results confirm that curing methods strongly influence the sensory quality of flue-cured tobacco.

**Table 2:** Comparison of the sensory qualities of tobacco leaves treated with different curing methods.

Quality Characteristic	TR	TZ	TC	CK
Aroma (36)	25.77±0.25a	24.93±0.31b	25.00±0.10b	25.43±0.31ab
Smoke (18)	12.93±0.12a	12.40±0.26b	12.93±0.23a	12.70±0.00ab
Taste (54)	37.03±0.12a	35.40±0.36b	36.70±0.20a	36.67±0.25a
Total score (108)	75.73±0.47a	72.73±0.90b	74.63±0.49a	74.80±0.52a

* Different letters indicate significant difference (P < 0.05) according to Tukey’s multiple range test.

###  Changes in enzyme activities during processing

**Fig. 4:**
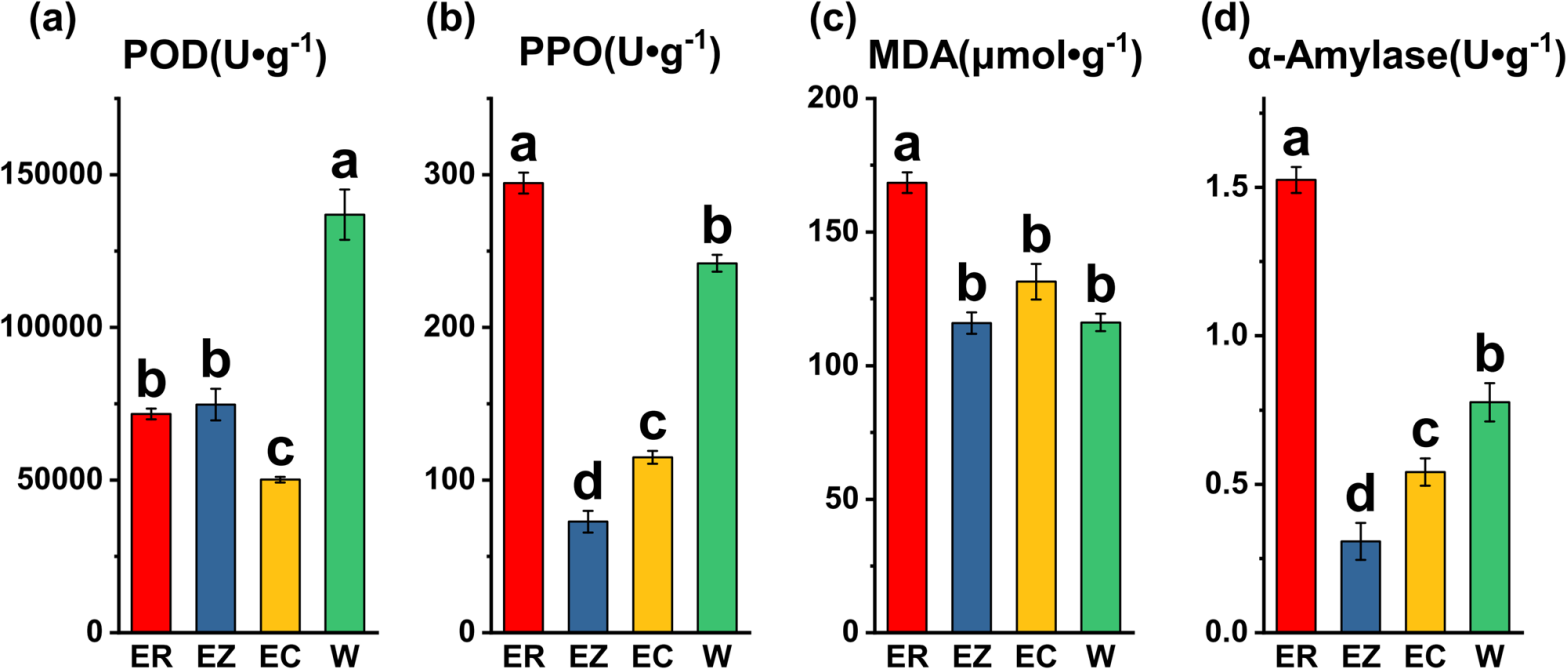
Enzyme activities in tobacco leaves after W, ER, EZ, and EC. (**a**) POD; (**b**) PPO; (**c**) MDA; (**d**) α-amylase. Different letters indicate a significant difference (*P* < 0.05) according to Tukey’s multiple range test.

Lipid peroxidation determines both the appearance and the abundance of polyphenolic compounds in flue-cured tobacco. Notably, PPO serves as the main catalyst, POD provides protection, and MDA indicates oxidative stress. Enzyme assays after ER, EZ, and EC revealed distinct activity patterns. POD activity increased in W and decreased after subsequent pretreatments; ER and EZ showed no significant difference, while EC had the lowest POD activity (Fig. 4a). PPO activity was highest in ER and was markedly reduced in EZ and EC; PPO activity in EC remained slightly higher than in EZ (Fig. 4b). MDA content was highest in ER, whereas EZ and EC did not differ significantly from W (Fig. 4c). Furthermore, α-amylase activity was highest in ER and lowest in EZ. EC retained significantly higher alpha-amylase activity than EZ (Fig. 4d).

Overall, steaming and pan-firing substantially reduced PPO activity. In contrast, rolling resulted in higher PPO and alpha-amylase activities and higher MDA content (Fig. 4).

### Changes in polyphenols between treatments

As shown in Table 3, polyphenol contents varied markedly among curing methods. Both TZ and TC significantly increased total polyphenol levels compared with TR, with TZ reaching the highest value (20.32 mg/g). CK (15.40 mg/g) was also significantly higher than TR (4.77 mg/g) and TC (13.16 mg/g), but lower than TZ. For individual compounds, TR exhibited the lowest concentrations of chlorogenic acid, rutin, and kaempferol. In contrast, scopolamine was significantly higher than in CK, TZ, and TC. TZ preserved markedly more chlorogenic acid and total polyphenols than TC, suggesting better protection of cellular and polyphenolic integrity during TZ, whereas TC caused partial degradation. Interestingly, CK contained higher chlorogenic acid and total polyphenols than TC, indicating that flue-curing can also favor polyphenol retention. Overall, relative to TR, all other curing methods increased polyphenol accumulation, with TZ proving most effective.

**Table 3:** Comparison of the polyphenol contents of tobacco leaves treated with different curing methods.

Polyphenols (mg/g)	TR	TZ	TC	CK
Chlorogenic acid	2.02±0.20d	11.21±0.60a	6.17±0.10c	8.74±0.27b
Scopoletin	0.22±0.01a	0.06±0.01d	0.11±0.02c	0.14±0.01b
Rutin	2.01±0.06d	8.27±0.07a	6.07±0.20b	5.74±0.26c
Kaempferol	0.51±0.01b	0.77±0.03a	0.80±0.03a	0.77±0.02a
Total polyphenol	4.77±0.12d	20.32±0.50a	13.16±0.29c	15.40±0.31b

* Different letters indicate significant difference (*P* < 0.05) according to Tukey’s multiple range test.

### Changes in macromolecular components between treatments

Starch and protein contents differed significantly among treatments (Table 4). Starch content ranked TR < CK < TC < TZ, with significant differences among treatments. Protein content also showed a broadly similar pattern. Notably, the starch content in TC was lower than in TZ.

**Table 4:** Comparison of tobacco leaf macromolecular substances treated with different curing methods.

Macromolecular substances	TR	TZ	TC	CK
Starch (%)	4.95±0.54d	10.09±0.64a	8.16±0.67b	6.79±0.23c
Protein (%)	5.32±0.25c	7.62±0.20a	8.03±0.50a	6.76±0.51b
Chlorophyll a (mg/g)	0.08±0.01b	0.06±0.01c	0.10±0.00b	0.12±0.02a
Chlorophyll b (mg/g)	0.04±0.01b	0.04±0.00ab	0.05±0.00a	0.04±0.01b
Carotenoids (mg/g)	0.09±0.01b	0.15±0.02a	0.11±0.01b	0.11±0.02b
Total chlorophyll (mg/g)	0.12±0.01bc	0.10±0.01c	0.15±0.00a	0.16±0.01a
Total plastid pigment (mg/g)	0.20±0.00b	0.25±0.01a	0.26±0.01a	0.27±0.02a

* Different letters indicate significant difference (*P* < 0.05) according to Tukey’s multiple range test.

Chlorophyll a, chlorophyll b, and carotenoids were the major plastid pigments (Table 4). TR showed the lowest total plastid pigment content, whereas TC, TZ, and CK did not differ significantly. Additionally, CK had the highest chlorophyll a content, and TZ had the lowest. TC had the highest chlorophyll b content, comparable to TZ, and higher than TR and CK. Total chlorophyll content was highest in TC and CK and lowest in TZ. Finally, TZ showed the highest carotenoid content, whereas TC, TR, and CK did not differ significantly.

### Changes in conventional chemical constituents between treatments

The chemical composition of tobacco leaves varied markedly with curing method (Table 5). The most pronounced effects were observed for total sugars, reducing sugars, total alkaloids, and total nitrogen. Importantly, total sugar content ranked TC > CK > TZ > TR, with TC showing the highest level. Reducing sugar content ranked TR > CK > TC > TZ, with TR showing the highest level. Total alkaloid content ranked CK > TZ > TR > TC, with TC and TR showing the strongest reductions. In contrast, Cl and K contents remained unaffected. Moreover, chemical balance indices varied among treatments. The reducing sugar/total sugar ratio was highest in TR (17.39) and lowest in TZ (9.62), with no significant difference between TC and CK. The total nitrogen/total alkaloid ratio peaked in TC (0.98) and was lowest in CK (0.78), while TR and TZ showed intermediate values.

**Table 5:** Comparison of the Chemical constituents of tobacco leaves treated with different curing methods.

Chemical constituents (%)	TR	TZ	TC	CK
Total sugar	29.31±0.20c	29.93±0.45c	32.88±0.60a	31.50±0.58b
Reducing sugar	28.50±0.10a	17.31±0.10d	20.81±0.80c	24.36±0.79b
Total alkaloids	1.64±0.02c	1.80±0.03b	1.59±0.01c	2.02±0.09a
Cl	1.40±0.04a	1.39±0.04a	1.38±0.03a	1.37±0.03a
K	2.02±0.02a	2.13±0.10a	2.10±0.05a	2.06±0.03a
Total Nitrogen	1.46±0.04b	1.53±0.05ab	1.56±0.02a	1.58±0.04a
Sugar/Alkaloids	17.39±0.15a	9.62±0.22c	13.09±0.42b	12.07±0.98b
Nitrogen/Alkaloids	0.89±0.01b	0.85±0.01b	0.98±0.02a	0.78±0.03c
Reducing Sugar/Total Sugar	0.97±0.01a	0.58±0.01d	0.63±0.04c	0.77±0.11b

* Different letters indicate significant difference (*P* < 0.05) according to Tukey’s multiple range test.

Together, these results demonstrate that the curing method substantially alters carbohydrate and nitrogen metabolism in tobacco leaves, whereas Cl and K remain largely stable.

### VOCs and multivariate analysis

**Fig. 5:**
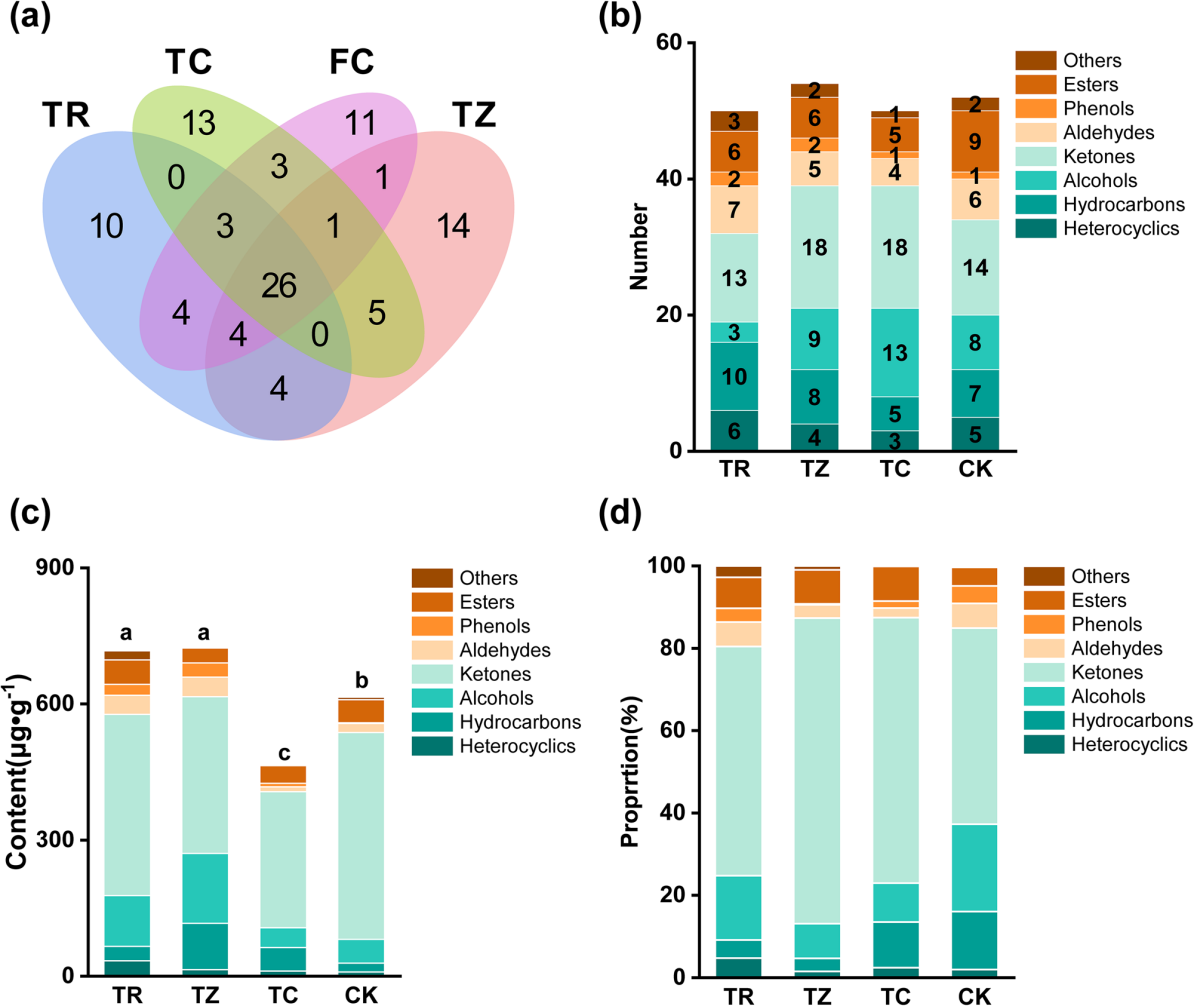
Overview of aroma compounds in tobacco leaves using different curing methods. (**a**) Venn plot; (**b**) classification of VOCs in 4 treatments; (**c**) The contents of various VOCs (excluding neophytadiene) in 4 treatments; (**d**) The proportion of VOCs. Different letters indicate a significant difference (*P* < 0.05) according to Tukey’s multiple range test.

GC-MS analysis identified 99 VOCs across tobacco leaves subjected to four curing methods (Table S1). TR, TZ, TC, and CK contained 51, 55, 51, and 53 compounds, respectively. Additionally, 26 VOCs were shared, while 10, 14, 13, and 11 were unique to TR, TZ, TC, and CK (Fig. 5a), indicating that curing substantially reshaped the VOC composition. Classification revealed that TR, TZ, TC, and CK contained 13, 18, 18, and 14 ketones; 3, 9, 13, and 8 alcohols; 10, 8, 5, and 7 hydrocarbons; 6, 6, 5, and 9 esters; 7, 5, 4, and 6 aldehydes; and 6, 4, 3, and 5 heterocyclics, respectively. Phenols and other minor groups were detected in smaller numbers (Fig. 5b).

Curing also altered the VOC’s relative abundance (Fig. 5c). TR and TZ produced the highest total VOC levels, while CK was significantly lower but still exceeded TC. Despite the relatively low total VOC content, CK accumulated the highest ketone levels. Notably, TZ was enriched in alcohols, TC in hydrocarbons, and TR in heterocyclics, esters, and other minor compounds (Table S1). Across all treatments, ketones dominated, followed by alcohols. Furthermore, CK exhibited the highest ketone proportion (74.17 %), whereas TZ had the lowest (47.63 %) but the highest alcohol proportion (21.23 %). CK contained the lowest proportion of alcohols (8.48 %) (Fig. 5d).

Overall, curing methods affected the total abundance of VOCs and the compositional balance among VOC classes.

**Fig. 6:**
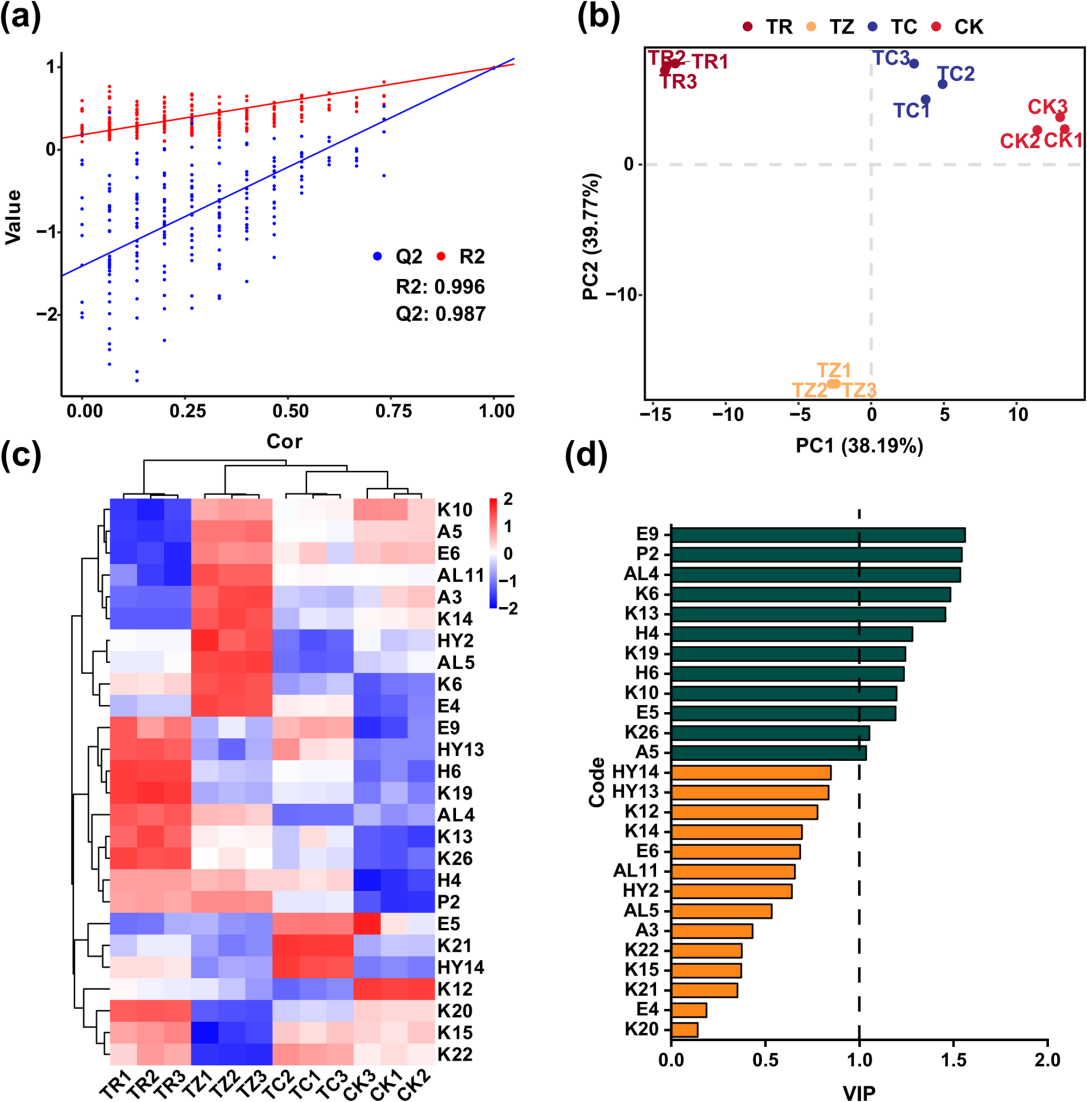
Analysis of 26 shared VOCs. (**a**) Loading plot of PLS-DA analysis in 4 treatments; (**b**) Score plot of PLS-DA analysis in 4 treatments; (**c**) Heatmap of 26 shared VOCs in 4 treatments; (**d**) Variable Importance in Projection (VIP) scores of VOCs.

To compare the shared VOC profiles, partial least squares discriminant analysis (PLS-DA) and heatmap visualization were applied to the 26 VOCs shared across the four curing methods (Fig. 6c). Distinct clustering patterns were evident for each group (Fig. 6b), indicating marked divergence in the abundance of shared VOCs. PC1 and PC2 explained 38.19% and 39.77% of the variance, respectively, and clearly separated the treatments, underscoring significant compositional differences. Model validation by permutation testing (n = 200) yielded Q^2^ = 0.967 and R^2^ = 0.996, confirming the robustness of the PLS-DA model (Fig. 6a).

Variable importance in projection (VIP) analysis identified 12 VOCs as key discriminators among treatments (Fig. 6d): dibutyl phthalate (E9), 2,4-Di-tert-butylphenol (P2), phenylacetaldehyde (AL4), 2,6,6-trimethyl-2-cyclohexene-1,4-dione (K6), damascone (K13), 2-acetylpyrrole (H4), 4,7,9-megastigmatrien-3-one1 (K19), indole (H6), 1-(4-hydroxy-3-methylphenyl)ethenone (K10), dihydroactinidiolide (E5), farnesyl acetone (K26), and phenethyl alcohol (A5). Importantly, these compounds have been previously linked to an odor, highlighting their functional importance in flavor formation.

The 12 VOCs selected based on VIP > 1 are presented in Table 6. Further investigation revealed that 8 of these substances have well-defined aromatic properties. Interestingly, three of them were present at significantly higher levels in the TR treatment compared to the other treatments. For example, indole can impart floral notes at low concentrations; 2-acetylpyrrole exhibits a nutty character; and phenylacetaldehyde produces a hyacinth-like aroma. In the TZ treatment, the contents of 2,6,6-Trimethyl-2-cyclohexene-1,4-dione and farnesyl acetone were significantly higher than in the other treatments, with these two compounds known for their distinct sweet and floral-ether aromas, respectively. Additionally, the dihydroactinidiolide content was significantly higher in the TZ and TC treatments than in the TR and TZ treatments, and a notable musk coumarin aroma characterizes this compound. Overall, the TR treatment exhibited higher levels of several VOCs. Thus, variations in the concentrations of these compounds may play a pivotal role in influencing the sensory characteristics of tobacco leaves during evaluation.

**Table 6.** Twelve key aroma components with VIP > 1 in different treatments (μg·g⁻^1^).

Code	Volatile compounds	CAS	Odor description^a^	TR	TZ	TC	CK
E9	dibutyl phthalate	84-74-2	–	37.32a	15.70b	12.40c	6.62d
P2	2,4-di-tert-butylphenol	96-76-4	–	23.22b	29.80a	7.85c	1.47d
AL4	phenylacetaldehyde	122-78-1	hyacinth aroma	31.41a	18.44b	6.20c	8.26c
K6	2,6,6-Trimethyl-2-cyclohexene-1,4-dione	1125-21-9	sweet aroma	1.90b	3.18a	1.26c	1.00d
K13	damascone	23726-91-2	fruity aroma	61.92a	45.73b	43.64b	31.28c
H4	2-acetylpyrrole	1072-83-9	nutty aroma	4.10a	3.49b	3.06b	0.87c
K19	4,7,9-megastigmatrien-3-one1	38818-55-2	–	22.81a	4.86c	6.58b	4.10c
H6	indole	120-72-9	floral aroma	20.28a	5.53c	7.11b	3.45d
K10	1-(4-hydroxy-3-methylphenyl) ethanone	876-02-8	–	1.25c	3.78a	2.71b	3.78a
E5	dihydroactinidiolide	17092-92-1	musk coumarin aroma	5.40b	5.54b	7.59a	7.10a
K26	farnesyl acetone	1117-52-8	flower ether aroma	12.16c	46.35a	29.98b	36.58b
A5	phenethyl alcohol	60-12-8	rose aroma	2.58d	10.02a	5.68c	6.86b

^a^Odor description referred to the database (The Good Scents Company Information System, http://www.thegoodscentscompany.com/). “–” Indicates not retrieved. Tukey’s HSD test was used for multiple comparisons. Different letters indicate significant differences among treatments (*p* < 0.05).

### Correlation analysis of chemical components and sensory attributes

**Fig. 7:**
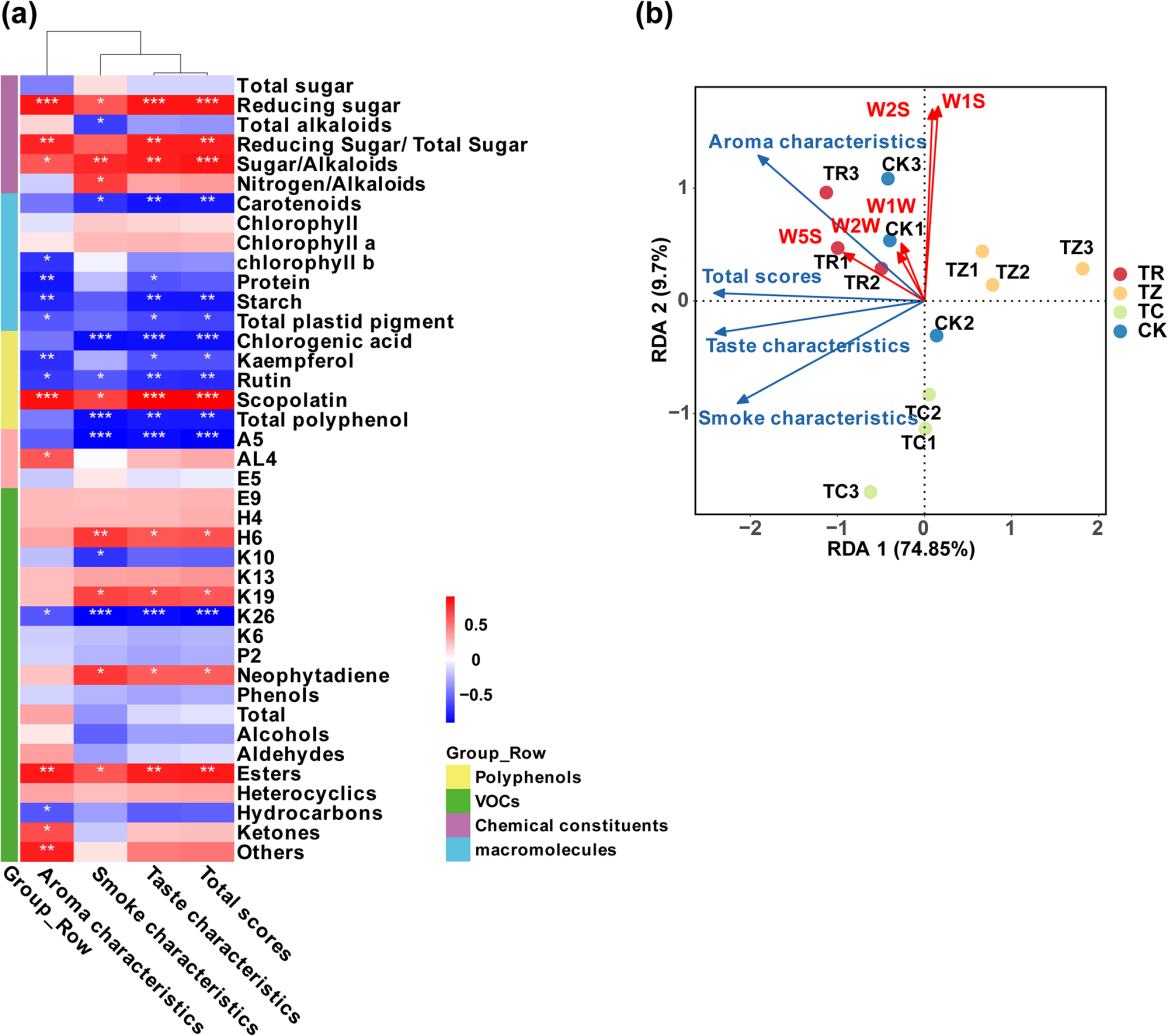
Correlation analysis between sensory quality and substances. (**a**) Heatmap showing Spearman’s rank correlations between the relative contents of VOCs, polyphenolic substances, major chemical components, and macromolecular constituents and sensory quality scores, * *P* < 0.05; ** *P* < 0.01; *** *P* < 0.001; (**b**) RDA of the relationships between E-nose sensor responses and sensory quality attributes.

To assess the contribution of polyphenolic compounds, macromolecules, major chemical constituents, and VOCs to sensory quality, Spearman’s correlation analysis was performed and visualized as a heat map (Fig. 7a). Scopoletin, the reducing sugar/total sugar ratio, the reducing sugar/total alkaloid ratio, esters, and reducing sugars were strongly and positively associated with aroma, smoke, taste, and total scores. Moreover, ketones, other aroma components, and AL4 also correlated positively with aroma. The total nitrogen/total alkaloids ratio was positively related to smoke scores, while H6, neophytadiene, and K19 were positively associated with smoke, taste, and total scores.

In contrast, rutin and K26 were negatively correlated with all sensory indices. Nicotine and K10 correlated negatively only with smoke characteristics. Starch, kaempferol, and total plastid pigments showed significant negative associations with aroma, taste, and total scores. In contrast, A5, K26, carotenoids, chlorogenic acid, and total polyphenols were negatively correlated with smoke, taste, and total scores. Chlorophyll b and hydrocarbons were negatively associated with aroma, and proteins were inversely related to aroma and taste characteristics.

Redundancy analysis (RDA) was conducted to relate the responses of differential E-nose sensors (W5S, W2W, W1W, W2S, W1S) to the main sensory dimensions (aroma, smoke, and taste characteristics) and the total score. The model was significant and well-explained: RDA explained 84.55% of the total variance (PC1 = 74.85%, PC2 = 9.7%, DCA1 = 0.00193, F = 7.71, Pr = 0.004), clearly separating the curing treatments (Fig. 7b). W1W, W2W, and W5S aligned positively with aroma characteristics and total scores, indicating they are key drivers of improved flavor quality. W2S and W1S were positively aligned with aroma but had minor effects on total scores, and their negative alignment with taste and smoke traits suggested a potential adverse influence.

Collectively, these analyses highlight scopoletin, the nitrogen/alkaloid ratio, reducing sugars, esters, and neophytadiene as key positive drivers of tobacco sensory quality, whereas rutin, K26, starch, proteins, and chlorogenic acid act as major constraints. The E-nose model underscores its potential as a rapid and reliable tool for predicting smoking quality and guiding curing strategies.

## Discussion

Findings from this study demonstrate that multiple tea-inspired curing methods are feasible for tobacco leaf curing. Their overall sensory performance was comparable to, or even superior to, that of conventional flue-curing, indicating that fresh tobacco leaves can be processed using more than one viable method. Consistent with this finding, PCA of electronic nose data and radar plots clearly distinguished the four treatments, and the responses of key sensors (W1W, W2W, W5S) were correlated with aroma sensory scores. This not only indicates the formation of distinct flavor styles but also underscores the potential of electronic nose technology for the quantitative evaluation of the sensory quality of tobacco leaf.

Among all treatments, TR exhibited the most pronounced sensory advantage, likely due to the cellular disruption induced by rolling and the subsequent enhancement of enzymatic reactions. Rolling markedly disrupted cellular compartmentalization, allowing enzymes and substrates to come into contact in the presence of oxygen, thereby establishing a highly reactive redox environment during the fermentation stage^34^. In this study, the activities of polyphenol oxidase (PPO) and α-amylase increased significantly and synchronously after rolling (Figures 4b and d). This indicates the coordinated activation of polyphenol oxidation and starch hydrolysis pathways^35^, providing conditions for the subsequent degradation of macromolecules and the conversion of aroma precursors^36^.

Excessively high starch content is generally considered unfavorable for the sensory quality of tobacco leaves^37^. The lower starch level observed in the TR treatment is likely attributed to the activation of α-amylase following rolling. During fermentation, starch is enzymatically hydrolyzed into maltose and subsequently converted into D-glucose by maltase (malZ), partially explaining the relatively lower total sugar but higher reducing sugar content detected in TR^38,39^. In this study, reducing sugar content was strongly positively correlated with various sensory scores, consistent with findings from previous research^40^. The degradation of proteins increased the availability of amino acids, thereby providing sufficient precursors for the formation of aromatic compounds such as phenylacetaldehyde and indole^41–44^. Moreover, the significantly elevated levels of phenylacetaldehyde and indole in the TR treatment (Table 6), which correspond to its lowest protein content (Table 4), indicate that the rolling-activated enzymatic activities enhanced downstream metabolism via the shikimate pathway. In addition, Strecker degradation of amino acids may have further contributed to the increased phenylacetaldehyde formation. Meanwhile, the relatively higher drying temperature likely promoted Maillard reactions between reducing sugars and amino acids, leading to increased formation of compounds such as 2-acetylpyrrole^45^.

Carotenoids are degraded during fermentation through two principal pathways: enzymatic cleavage catalyzed by carotenoid cleavage dioxygenases (CCDs), leading to the formation of key aroma compounds such as linalool and damascone, and non-enzymatic oxidative cleavage mediated by reactive oxygen species (ROS) and oxidants such as hydrogen peroxide (H₂O₂)^46^. In this study, the fermentation process of TR was similar to that of black tea, resulting in a significant increase in the levels of 4,7,9-megastigmatrien-3-one 1 (K19) and damascene (H4) (Table 6). Although no specific aroma descriptor has been assigned to K19, its content exhibited a significant positive correlation with the overall sensory score (Figure 7a), indicating its important contribution to the overall flavor profile^47^. However, TR treatment did not significantly elevate the levels of other isoprenoid compounds, such as linalool (A4) and β-ionone (K16), a phenomenon that may be attributed to physiological differences between tobacco leaves and tea leaves (Table S1). Furthermore, neophytadiene was the most abundant volatile compound detected across treatments. It showed a significant positive correlation with sensory indices (Figure 7a), consistent with its proposed role as an aroma-related carrier compound^48–50^. The fermentation process of TR likely accelerated the degradation of chlorophyll into phytol^51^, which was subsequently converted to neophytadiene. This proposed mechanism explains the concurrent significant decreases in chlorophyll a content and increases in neophytadiene content observed in TR (Table 4), a compositional shift that positively influenced sensory quality.

In contrast, TR, TZ, and TC involved high-temperature treatment before fermentation, which rapidly inactivated PPO and effectively suppressed polyphenol oxidation^52^. As a result, TZ and TC exhibited significantly higher polyphenol content than TR (Table 3), confirming that thermal inactivation of PPO is an effective strategy for enhancing polyphenol retention. However, premature enzyme inactivation may also have limited the degradation of macromolecules and restricted the subsequent conversion of aroma precursors into volatile compounds during fermentation^53,54^. Accordingly, starch, protein, and plastid pigment contents in TZ and TC were significantly higher than those in TR (Table 4), and reducing sugar levels were significantly lower (Table 5). In addition, key aroma compounds such as phenylacetaldehyde and K19 were present at substantially lower levels in TZ and TC than in TR (Table 6). These suggest that although TZ and TC exhibit advantages in polyphenol preservation, they may have inherent limitations in their efficiency for macromolecular degradation and aroma formation. While polyphenol retention is important, elevating a single component alone is unlikely to improve overall sensory quality substantially.

Although TZ and TC showed comparable performance in polyphenol retention, clear differences were observed in aroma formation and sensory performance (Table 2), highlighting the importance of heating mode. Steaming is a relatively uniform moist-heat process that allows the entire leaf to rapidly reach a high temperature, resulting in consistent and thorough enzyme inactivation^55^. In contrast, pan-firing is a non-uniform dry-heat treatment that creates spatial heterogeneity in heat input across different regions of the leaf^56^. Under such conditions, certain heat-tolerant hydrolytic enzymes may be partially retained in localized areas during TC processing, thereby continuing to participate in substrate transformation. This may explain why the reducing sugar content in TC was significantly higher than that in TZ (Table 5). Moreover, the elevated temperatures during EC likely promoted the volatilization of low-boiling off-flavor compounds (e.g., grassy notes)^57^. This may likely account for the lower total VOC content observed in TC. Therefore, the combined effects of residual enzymatic activity and physical removal of undesirable volatiles may have contributed to TC’s superior sensory performance compared with TZ.

Although TZ exhibited relatively high total VOCs and polyphenol contents, its sensory evaluation score was the lowest among all treatments. This may be attributed to higher levels of plastid pigments, starch, and proteins, which can generate off-notes such as miscellaneous odors, scorched notes, or burnt-feather–like aromas during smoking, thereby masking potential aroma advantages^58,59^. This suggests that a prerequisite for high-quality tobacco leaves is a relatively low content of macromolecular substances, which is consistent with previous research findings. Furthermore, excessively high polyphenol levels may also negatively impact sensory evaluation^60^.

CK did not exhibit extreme advantages or disadvantages in polyphenol content, sugar–alkaloid balance, volatile aroma composition, or sensory attributes, yet it maintained a relatively stable overall sensory quality. This indicates that improvements in tobacco quality do not depend on the marked enhancement of any single component but rather on the coordinated balance among multiple quality-related factors^61^.

Overall, TR, TZ, and TC represent three quality formation pathways with distinct emphases. TR, through rolling-induced activation of enzymatic reactions, reduced polyphenol levels but achieved extensive macromolecule degradation, enhanced amino acid metabolic pathways, and synergistic accumulation of aroma compounds, resulting in a pronounced sensory advantage. In contrast, TZ and TC effectively preserved polyphenols through high-temperature enzyme inactivation but differed in their capacities for macromolecule degradation and aroma formation. It is noteworthy that these three modulation processes may benefit from adopting the “open modulation” concept derived from tea processing. By introducing enzyme-active microorganisms capable of degrading macromolecules, the sensory quality could potentially be further improved^62^. Among them, the TZ process may exhibit greater application potential due to its intrinsic characteristics. Similar strategies have achieved encouraging progress in tea processing.

This study demonstrates that purposeful combination and regulation of key processing steps can direct a single tobacco raw material toward differentiated flavor styles. This tea-inspired curing methods concept breaks through the limitations of traditional single curing methods and provides a novel technical framework for directional quality regulation and process innovation in tobacco.

It should be noted that this study still has certain limitations. First, volatile aroma compounds were primarily analyzed based on relative quantification using GC-MS, and absolute quantification of key VOCs was not performed. The electronic nose was used to characterize overall odor responses of unburned tobacco leaves at ambient temperature, and cannot directly reflect the composition of mainstream smoke under combustion conditions. In addition, this study investigated only a single tobacco cultivar, and the effects of cultivar differences on processing outcomes require further exploration.

Accordingly, future studies may systematically investigate processing parameters across multiple growing regions, cultivars, and leaf positions to clarify the applicability of different processing routes and further improve quality performance. In parallel, integrating multi-omics approaches to elucidate aroma formation mechanisms across different processing pathways will further advance theoretical understanding of quality formation and regulation during tobacco processing.

In the manuscript, AI tools were not used to analyze data or draw insights as part of the research process; they were only used to correct grammar and improve readability.

## Electronic Supplementary Material

Below is the link to the electronic supplementary material.

Supplementary Information

## Data Availability

The findings of this study are available from the corresponding author upon reasonable request.
