# Supplementary material for: Tea-inspired curing modulates chemical composition, volatile aroma, and sensory quality of flue-cured tobacco leaves
Source: Sci Rep. 2026 Apr 14;16:22764. doi: 10.1038/s41598-026-48595-z (PMC13385958; doi:10.1038/s41598-026-48595-z)
Supplement: Supplementary file 1 — Supplementary Information [file 41598_2026_48595_MOESM1_ESM.docx]

Table S1 VOCs in different treatments.

| Category | Code | Name | CAS | RI | RT(min) | TR( μg·g⁻¹) | TZ( μg·g⁻¹) | TC( μg·g⁻¹) | CK( μg·g⁻¹) |
| --- | --- | --- | --- | --- | --- | --- | --- | --- | --- |
| Heterocyclics | H1 | 2-acetylfuran | 1192-62-7 | 878 | 6.283 | 3.11a | \ | 1.14b | 2.88a |
|  | H2 | 2-amylfuran | [3777-69-3](https://www.chemsrc.com/baike/196939.html) | 1040 | 10.825 | 3.01a | 1.59b | \ | 0.84c |
|  | H3 | 2-pent-2-enylfuran | 82550-42-3 | 1048 | 11.546 | 2.12 | \ | \ | \ |
|  | H4 | 2-acetylpyrrole | 1072-83-9 | 1035 | 15.979 | 4.10a | 3.49b | 3.06b | 0.87c |
|  | H5 | [coumaran](https://www.chemsrc.com/en/cas/496-16-2_1194361.html) | 496-16-2 | 1036 | 28.067 | 1.37a | \ | \ | 1.30a |
|  | H6 | indole | 120-72-9 | 1174 | 32.862 | 20.28a | 5.53c | 7.11b | 3.45d |
|  | H7 | 3-acetylindole | 703-80-0 | 1854 | 63.374 | \ | 3.78 | \ | \ |
|  | Total |  |  |  |  | 33.97a | 14.39b | 11.31bc | 9.33c |
| Hydrocarbons | HY1 | trans-5-decene | 7433-56-9 | 1023 | 7.322 | 2.96a | \ | \ | 2.65a |
|  | HY2 | 5-methylhex-4-enoic acid | 5636-65-7 | 1000 | 9.374 | 7.21b | 15.98a | 3.89d | 6.42cd |
|  | HY3 | 1-decene | 872-05-9 | 1005 | 11.063 | \ | \ | 0.68 | \ |
|  | HY4 | trans-4-decene | 19398-89-1 | 1023 | 11.205 | \ | \ | \ | 0.68 |
|  | HY5 | pentylcyclopentane | 3741-00-2 | 1058 | 14.201 | 0.82b | 2.58a | \ | 0.69b |
|  | HY6 | 5-methylundecane | 1632-70-8 | 1150 | 23.558 | \ | 1.39 | \ | \ |
|  | HY7 | [2,6-dimethyldecane](https://www.chemsrc.com/en/cas/13150-81-7_122290.html) | 13150-81-7 | 1086 | 23.633 | 0.62 | \ | \ | \ |
|  | HY8 | dodecane | 112-40-3 | 1214 | 26.937 | 1.43b | 2.43a | \ | \ |
|  | HY9 | [cyclododecane](https://www.chemsrc.com/en/cas/294-62-2_1191439.html) | 294-62-2 | 1439 | 29.821 | 1.00 | \ | \ | \ |
|  | HY10 | 1-heptadecene | 6765-39-5 | 1680 | 52.316 | \ | 5.13 | \ | \ |
|  | HY11 | (-)-alpha-cedrene | 469-61-4 | 1403 | 52.489 | \ | \ | \ | 2.80 |
|  | HY12 | 3-methylheptadecane | 6418-44-6 | 1989 | 60.455 | 2.71b | \ | 3.48a | 0.72c |
|  | HY13 | 1-nonadecene | 18435-45-5 | 1900 | 61.288 | 9.47a | 5.57c | 7.68b | 5.43c |
|  | HY14 | neophytadiene | [504-96-1](https://www.chemsrc.com/baike/467.html) | 0 | 63.005 | 2334.43a | 1732.70b | 2152.91a | 1457.71c |
|  | HY15 | heptadecane | [629-78-7](https://www.chemsrc.com/baike/584755.html) | 1711 | 65.559 | 1.65 | \ | \ | \ |
|  | HY16 | [cembrene](https://www.chemsrc.com/en/cas/1898-13-1_166408.html) | [1898-13-1](https://www.chemsrc.com/baike/166408.html) | 2072 | 66.776 | 3.86b | 42.03a | \ | \ |
|  | HY17 | squalene | [111-02-4](https://www.chemsrc.com/baike/165516.html) | 2914 | 101.055 | \ | 26.86b | 35.87a | \ |
|  | Total (Except neophytadiene) | |  |  |  | 31.73c | 101.99a | 51.60b | 19.39d |
| Alcohols | A1 | 5-methyl-2-furanmethanol | 3857-64-3 | 975 | 8.245 | \ | 1.16 | \ | \ |
|  | A2 | 2-n-propyl-1-pentanol | 58175-57-8 | 995 | 13.801 | \ | \ | 1.65 | \ |
|  | A3 | benzyl alcohol | 100-51-6 | 1036 | 13.900 | 7.14c | 14.26a | 8.68c | 10.38b |
|  | A4 | linalool | 78-70-6 | 1082 | 19.185 | \ | 2.53a | 2.33a | \ |
|  | A5 | phenethyl alcohol | 60-12-8 | 1136 | 19.786 | 2.58d | 10.02a | 5.68c | 6.86b |
|  | A6 | (s)-(-)-α-terpineol | 10482-56-1 | 1143 | 26.140 | \ | \ | 1.34 | \ |
|  | A7 | 3,7-dimethyl-2,6-octadien-1-ol | [624-15-7](https://www.chemsrc.com/baike/4201.html) | 1228 | 30.459 | \ | \ | 2.18 | \ |
|  | A8 | (e)-1-cyclohexylbut-2-en-1-ol | [79646-42-7](https://www.chemsrc.com/baike/742360.html" \t "https://www.chemsrc.com/searchResult/(E)-1-cyclohexylbut-2-en-1-ol/_blank) | 1249 | 32.375 | \ | \ | \ | 1.57 |
|  | A9 | 4,8-dimethylnon-7-en-2-ol | [40596-76-7](https://www.chemsrc.com/baike/294733.html) | 1229 | 34.062 | \ | 2.57a | 1.35b | \ |
|  | A10 | 2-methyl-2-(4-methyl-3-pentenyl)-cyclopropanemethanol | [98678-70-7](https://www.chemsrc.com/baike/1466090.html" \t "https://www.chemsrc.com/searchResult/2-Methyl-2-(4-methyl-3-pentenyl)-cyclopropanemethanol/_blank) | 1280 | 35.047 | \ | \ | \ | 0.58 |
|  | A11 | farnesol | [4602-84-0](https://www.chemsrc.com/baike/249701.html) | 1710 | 48.044 | \ | 1.86a | 0.75b | 2.12a |
|  | A12 | nerolidol | [7212-44-4](https://www.chemsrc.com/baike/406567.html) | 1677 | 50.316 | \ | 2.14 | \ | \ |
|  | A13 | [2-hexyl-1-decanol](https://www.chemsrc.com/en/cas/2425-77-6_1148884.html) | [2425-77-6](https://www.chemsrc.com/baike/1148884.html) | 1790 | 56.084 | \ | 2.04a | 1.56b | \ |
|  | A14 | [1-heptadecanol](https://www.chemsrc.com/en/cas/1454-85-9_671030.html) | [1454-85-9](https://www.chemsrc.com/baike/671030.html) | 1954 | 56.392 | \ | \ | 1.76 | \ |
|  | A15 | 12-isopropyl-1,5,9-trimethyl-4,8,13-cyclotetradecatriene-1,3-diol | [7220-78-2](https://www.chemsrc.com/baike/1465756.html) | 2400 | 58.247 | \ | \ | \ | 0.81 |
|  | A16 | 11-methyl-1-dodecanol | [27458-92-0](https://www.chemsrc.com/baike/263980.html) | 1492 | 58.826 | \ | \ | 1.67 | \ |
|  | A17 | 3,7,11,15-tetramethyl-2-hexadecen-1-ol | [102608-53-7](https://www.chemsrc.com/baike/345696.html) | 2045 | 63.883 | \ | \ | 12.25a | 2.58b |
|  | A18 | isocembrol | [80126-41-6](https://www.chemsrc.com/baike/1418201.html) | 2211 | 74.786 | 102.36a | 117.45a | \ | 27.26b |
|  | A19 | [geranylgeraniol](https://www.chemsrc.com/en/cas/24034-73-9_330983.html) | [7614-21-3](https://www.chemsrc.com/baike/200967.html) | 2192 | 85.398 | \ | \ | 2.64 | \ |
|  | Total | |  |  |  | 112.08b | 154.01a | 43.86c | 52.15c |
| Ketones | K1 | 4-hydroxy-2-butanone | [590-90-9](https://www.chemsrc.com/baike/401110.html) | 798 | 7.293 | \ | \ | 5.09 | \ |
|  | K2 | 6-methyl-2-heptanone | 9[28-68-7](https://www.chemsrc.com/baike/244389.html) | 888 | 8.395 | \ | 1.00a | \ | 1.39a |
|  | K3 | 2-methyl-1-hepten-6-one | [10408-15-8](https://www.chemsrc.com/baike/1121918.html) | 933 | 10.441 | \ | 17.38 | \ | \ |
|  | K4 | 6-methylhept-5-en-2-one | [110-93-0](https://www.chemsrc.com/baike/509694.html) | 938 | 10.509 | \ | \ | 2.86b | 10.31a |
|  | K5 | acetophenone | [98-86-2](https://www.chemsrc.com/baike/33377.html) | 1043 | 16.205 | \ | 1.76 | \ | \ |
|  | K6 | 2,6,6-trimethyl-2-cyclohexene-1,4-dione | [1125-21-9](https://www.chemsrc.com/baike/245522.html) | 1268 | 22.283 | 1.90b | 3.18a | 1.26c | 1.00d |
|  | K7 | 2,2,6-trimethyl-1,4-cyclohexanedione | [20547-99-3](https://www.chemsrc.com/baike/1044836.html) | 1258 | 24.206 | \ | \ | 0.36 | \ |
|  | K8 | (5s)-5-(hydroxymethyl)oxolan-2-one | [32780-06-6](https://www.chemsrc.com/baike/248099.html) | 1129 | 25.217 | \ | \ | 2.80 | \ |
|  | K9 | 3-ethyl-4-methylpyrrole-2,5-dione | [20189-42-8](https://www.chemsrc.com/baike/1028189.html) | 1195 | 28.992 | \ | \ | 0.71 | \ |
|  | K10 | 1-(4-hydroxy-3-methylphenyl)ethanone | [876-02-8](https://www.chemsrc.com/baike/1154218.html" \t "https://www.chemsrc.com/searchResult/1-(4-Hydroxy-3-methylphenyl)ethanone/_blank) | 1363 | 34.207 | 1.25c | 3.78a | 2.71b | 3.78a |
|  | K11 | (e)-3-nonen-2-one | [18402-83-0](https://www.chemsrc.com/baike/88289.html)-3-nonen-2-one/_blank) | 1060 | 36.263 | \ | \ | \ | 0.43 |
|  | K12 | d-Solanone | [1937-54-8](https://www.chemsrc.com/baike/1298928.html) | 1296 | 37.774 | 150.88b | 130.47b | 105.91c | 265.79a |
|  | K13 | damascone | [23726-91-2](https://www.chemsrc.com/baike/752173.html) | 1440 | 38.866 | 61.92a | 45.73b | 43.64b | 31.28c |
|  | K14 | geranylacetone | [3796-70-1](https://www.chemsrc.com/baike/600196.html) | 1420 | 43.482 | 14.58d | 32.37a | 19.34c | 22.15b |
|  | K15 | 1,3,7,7-tetramethyl-2-oxabicyclo[4.4.0]-5-decen-9-one | [20194-67-6](https://www.chemsrc.com/baike/1465037.html) | 1541 | 45.045 | 5.50a | 2.47c | 4.74b | 4.67b |
|  | K16 | β-ionone | [79-77-6](https://www.chemsrc.com/baike/164361.html) | 2009 | 45.212 | 1.44b | 5.44a | \ | \ |
|  | K17 | beta-ionone epoxide | [23267-57-4](https://www.chemsrc.com/baike/263464.html) | 2331 | 45.349 | \ | 3.86 | \ | \ |
|  | K18 | 2,5,5,8a-tetramethyl-2,3,4,4a,6,8-hexahydrochromen-7-one | [5835-18-7](https://www.chemsrc.com/baike/930499.html) | 1531 | 46.915 | 0.67 | \ | \ | \ |
|  | K19 | 4,7,9-megastigmatrien-3-one1 | [38818-55-2](https://www.chemsrc.com/baike/1204712.html) | 1545 | 49.817 | 22.81a | 4.86c | 6.58b | 4.10c |
|  | K20 | 4,7,9-megastigmatrien-3-one2 | [38818-55-2](https://www.chemsrc.com/baike/1204712.html) | 1545 | 49.817 | 88.12a | 11.10d | 24.19c | 41.34b |
|  | K21 | 4,7,9-megastigmatrien-3-one3 | [38818-55-2](https://www.chemsrc.com/baike/1204712.html) | 1545 | 50.851 | 4.78b | 4.21c | 6.85a | 4.51b |
|  | K22 | 4,7,9-megastigmatrien-3-one4 | [38818-55-2](https://www.chemsrc.com/baike/1204712.html) | 1545 | 53.359 | 33.09a | 16.40c | 34.65a | 28.95b |
|  | K23 | 3,5-di-tert-butyl-4-hydroxyacetophenone | [14035-33-7](https://www.chemsrc.com/baike/254560.html) | 1903 | 61.496 | \ | \ | 2.67 | \ |
|  | K24 | dihydro-gamma-ionone | [13720-12-2](https://www.chemsrc.com/baike/155144.html) | 2102 | 62.047 | \ | 12.68 | \ | \ |
|  | K25 | 1-oxaspiro[4.5]deca-6,9-diene-2,8-dione, 7,9-bis(1,1-dimethylethyl)- | [82304-66-3](https://www.chemsrc.com/baike/987701.html" \t "https://www.chemsrc.com/searchResult/1-Oxaspiro%255B4.5%255Ddeca-6%252C9-diene-2%252C8-dione%252C%25207%252C9-bis(1%252C1-dimethylethyl)-/_blank) | 2081 | 65.186 | \ | 2.51b | 5.33a | \ |
|  | K26 | farnesyl acetone | [1117-52-8](https://www.chemsrc.com/baike/509665.html) | 1902 | 65.684 | 12.16c | 46.35a | 29.98b | 36.58b |
|  | Total | |  |  |  | 399.11b | 345.55c | 299.67d | 456.28a |
| Aldehydes | AL1 | benzaldehyde | [100-52-7](https://www.chemsrc.com/baike/946834.html) | 982 | 8.801 | \ | 4.78 | \ | \ |
|  | AL2 | 5-methyl-2-furaldehyde | [620-02-0](https://www.chemsrc.com/baike/952378.html) | 920 | 8.831 | 2.72 | \ | \ | 2.19 |
|  | AL3 | [2-formyl-1H-pyrrole](https://www.chemsrc.com/en/cas/1003-29-8_376314.html) | [254729-95-8](https://www.chemsrc.com/baike/766033.html) | 988 | 12.251 | 0.58 | \ | \ | \ |
|  | AL4 | phenylacetaldehyde | [122-78-1](https://www.chemsrc.com/baike/829288.html) | 1081 | 14.572 | 31.41a | 18.44b | 6.20c | 8.26c |
|  | AL5 | nonanal | [124-19-6](https://www.chemsrc.com/baike/1102354.html) | 1104 | 19.555 | 4.01b | 14.74a | 1.61c | 3.60b |
|  | AL6 | (2E,6Z)-nona-2,6-dienal | [557-48-2](https://www.chemsrc.com/baike/246358.html" \t "https://www.chemsrc.com/searchResult/(2E%252C6Z)-nona-2%252C6-dienal/_blank) | 1120 | 23.195 | 2.47 | \ | \ | \ |
|  | AL7 | 2,4-dimethylbenzaldehyde | [15764-16-6](https://www.chemsrc.com/baike/80194.html) | 1208 | 24.575 | 0.58 | \ | \ | \ |
|  | AL8 | 1,3,4-trimethylcyclohex-3-enecarbaldehyde | [40702-26-9](https://www.chemsrc.com/baike/669084.html) | 1204 | 27.838 | \ | \ | 1.23a | 1.45a |
|  | AL9 | 5-hydroxymethylfurfural | [67-47-0](https://www.chemsrc.com/baike/1192568.html) | 1163 | 28.413 | \ | \ | \ | 2.81 |
|  | AL10 | undecanal | [112-44-7](https://www.chemsrc.com/baike/828385.html) | 1133 | 34.401 | \ | 0.89 | \ | \ |
|  | AL11 | decanal | [112-31-2](https://www.chemsrc.com/baike/584838.html) | 1402 | 27.194 | 0.59c | 4.57a | 1.68b | 1.63b |
|  | Total | |  |  |  | 42.35a | 43.42a | 10.73c | 19.94b |
| Phenols | P1 | phenol | [108-95-2](https://www.chemsrc.com/baike/1101388.html) | 901 | 9.946 | 0.72b | 1.17a | \ | \ |
|  | P2 | 2,4-Di-tert-butylphenol | [96-76-4](https://www.chemsrc.com/baike/114.html) | 1555 | 47.168 | 23.22b | 29.80a | 7.85c | 1.47d |
|  |  |  |  | Total |  | 23.94b | 30.97a | 7.85c | 1.47d |
| Esters | E1 | linalyl acetate | [115-95-7](https://www.chemsrc.com/baike/509673.html) | 1272 | 19.271 | 1.52a | \ | \ | 1.67a |
|  | E2 | (-)-(4S)-4,5-dihydro-4-hydroxyfuran-2(3H)-one | [5469-16-9](https://www.chemsrc.com/baike/1101014.html" \t "https://www.chemsrc.com/searchResult/(-)-(4S)-4%252C5-dihydro-4-hydroxyfuran-2(3H)-one/_blank) | 1013 | 23.282 | \ | \ | \ | 3.23 |
|  | E3 | ethyl benzoate | [93-89-0](https://www.chemsrc.com/baike/951478.html) | 1134 | 24.275 | \ | 1.55 | \ | \ |
|  | E4 | methyl salicylate | [119-36-8](https://www.chemsrc.com/baike/1150765.html) | 1281 | 25.752 | 1.18c | 3.63a | 1.66b | 0.75d |
|  | E5 | dihydroactinidiolide | [17092-92-1](https://www.chemsrc.com/baike/752955.html) | 1426 | 47.626 | 5.40b | 5.54b | 7.59a | 7.10a |
|  | E6 | diisobutyl phthalate | [84-69-5](https://www.chemsrc.com/baike/443263.html) | 1908 | 63.509 | 5.25b | 3.06c | 10.02a | 2.73c |
|  | E7 | methyl 3-(3,5-di-Tert-Butyl-4-Hydroxyphenyl)Propionate | [6386-38-5](https://www.chemsrc.com/baike/165144.html" \t "https://www.chemsrc.com/searchResult/Methyl%25203-(3%252C5-di-Tert-Butyl-4-Hydroxyphenyl)Propionate/_blank) | 2088 | 66.431 | \ | 3.42 | \ | \ |
|  | E8 | [methyl palmitate](https://www.chemsrc.com/en/cas/112-39-0_832310.html) | [112-39-0](https://www.chemsrc.com/baike/832310.html) | 1878 | 66.575 | 3.36c | \ | 7.45b | 21.16a |
|  | E9 | [dibutyl phthalate](https://www.chemsrc.com/en/cas/84-74-2_336203.html) | [84-74-2](https://www.chemsrc.com/baike/336203.html) | 2037 | 67.576 | 37.32a | 15.70b | 12.40c | 6.62d |
|  | E10 | methyl linolenate | [301-00-8](https://www.chemsrc.com/baike/674223.html) | 2101 | 76.595 | \ | \ | \ | 5.66 |
|  | E11 | vinyl Myristate | [5809-91-6](https://www.chemsrc.com/baike/122781.html) | 1769 | 59.524 | \ | \ | \ | 1.87 |
|  | Total |  |  |  |  | 54.04a | 32.89c | 39.13b | 50.79a |
| Other | O1 | 1-(3,4-dihydro-2H-pyrrol-5-yl)ethanone | [85213-22-5](https://www.chemsrc.com/baike/438.html" \t "https://www.chemsrc.com/searchResult/1-(3%252C4-dihydro-2H-pyrrol-5-yl)ethanone/_blank) | 932 | 6.738 | \ | 0.76 | \ | \ |
|  | O2 | [aniline](https://www.chemsrc.com/en/cas/62-53-3_954652.html) | [62-53-3](https://www.chemsrc.com/baike/954652.html) | 835 | 9.735 | \ | \ | 0.61 | \ |
|  | O3 | n-Methylaniline | [100-61-8](https://www.chemsrc.com/baike/830277.html) | 992 | 12.902 | \ | \ | \ | 0.46 |
|  | O4 | 1,2-diethyl-4-phenylbenzene | [61141-66-0](https://www.chemsrc.com/baike/1074915.html) | 1792 | 56.324 | 1.38 | \ | \ | \ |
|  | O5 | 2,3,6-trimethylnaphthalene-1,4-dione | [20490-42-0](https://www.chemsrc.com/baike/934302.html) | 1783 | 59.228 | 3.90b | 1.53c | \ | 5.38a |
|  | O6 | 1,4,6-trimethyl-1,2,3,4-tetrahydronaphthalene | [22824-32-4](https://www.chemsrc.com/baike/873769.html) | 1402 | 41.046 | 14.54 | \ | \ | \ |
|  | Total | |  |  |  | 19.83a | 2.29c | 0.61d | 5.84b |
|  | Total (Except neophytadiene) | | | | | 717.04a | 727.10a | 464.75c | 615.19b |

Note:”/” means that the substance has not been detected; Different letters in the same group mean significant difference at P<0.05 between treatments.
